# Geographic Separation of Domestic and Wild Strains of *Toxoplasma gondii* in French Guiana Correlates with a Monomorphic Version of Chromosome1a

**DOI:** 10.1371/journal.pntd.0003182

**Published:** 2014-09-18

**Authors:** Asis Khan, Daniel Ajzenberg, Aurélien Mercier, Magalie Demar, Stéphane Simon, Marie Laure Dardé, Qiuling Wang, Shiv Kumar Verma, Benjamin M. Rosenthal, Jitender P. Dubey, L. David Sibley

**Affiliations:** 1 Department of Molecular Microbiology, Washington University School of Medicine, St. Louis, Missouri, United States of America; 2 Centre National de Référence Toxoplasmose/Toxoplasma Biological Resource Center, Institut National de la Santé et de la Recherche Médicale, Unité Mixte de Recherche 1094, Neuroépidémiologie Tropicale, Laboratoire de Parasitologie-Mycologie, Faculté de Médecine, Université de Limoges, Limoges, France; 3 Laboratoire de Parasitologie-Mycologie, Centre Hospitalier de Cayenne and Faculté de Médecine, Equipe EA 3593, Ecosystèmes Amazoniens et Pathologie Tropicale, Université des Antilles et de la Guyane, Cayenne, French Guiana; 4 Animal Parasitic Disease Laboratory, Beltsville Agricultural Research Center, United States Department of Agriculture, Beltsville, Maryland, United States of America; Johns Hopkins Bloomberg School of Public Health, United States of America

## Abstract

**Background:**

Previous studies have stressed the genetic divergence and high pathogenicity of strains of *T. gondii* from French Guiana. Although strains from coastal, human adapted environments (so called anthropized) resemble those found in other regions of the Caribbean, strains collected from inland jungle environment are genetically quite diverse. To better understand the composition of these distinct strain types, we undertook a more in depth analysis of *T. gondii* strains from French Guiana including profiling of chromosome 1a (Chr1a), which is often shared as a single monomorphic haplotype among lineages that are otherwise genetically distinct.

**Methodology/Principal Findings:**

Comparison of intron sequences from selectively neutral genes indicated that anthropized strains were most closely related to clonal type III strains from North America, although wider RFLP analysis revealed that they are natural hybrids. In contrast, strains isolated from the jungle were genetically very diverse. Remarkably, nearly all anthropized strains contained the monomorphic version of Chr1a while wild stains were extremely divergent. The presence of the monomorphic Chr1a strongly correlated with greater transmission in domestic cats, although there were several exceptions, indicating that other factors also contribute. Anthropized strains also varied in their virulence in laboratory mice, and this pattern could not be explained by the simple combination of previously identified virulence factors, indicating that other genetic determinants influence pathogenicity.

**Conclusions/Significance:**

Our studies underscore the marked genetic separation of anthropized and wild strains of *T. gondii* in French Guiana and provide additional evidence that the presence of Chr1a is associated with successful expansion of widely different lineages within diverse geographic areas. The predominance of Chr1a among strains in the anthropized environment suggests that it may confer an advantage for transmission in this environment, and thus potentially contribute to the spread of pathogenecity determinants.

## Author Summary

*Toxoplasma gondii* is a widespread parasite of animals that is easily transmitted to humans. Previous studies have shown that human infections in jungle areas of French Guiana are often quite severe, unlike most human infections that are characterized by mild symptoms in healthy adults. Here we characterized the genetic makeup of strains from French Guiana and confirm that while genetically homogeneous strains exist in anthropized environments, highly divergent and pathogenic isolates are found in jungle environments. The geographic separation of strain types is also mirrored in conserved genomic regions, including a monomorphic version of chromosome 1a (Chr1A), which has previously been associated with the spread of different lineages around the world. Strains harboring the monomorphic Chr1a showed greater potential for transmission in domestic cats, which may contribute to their prevalence in anthropized environments. Our findings also revealed large differences in acute virulence of French Guiana isolates in the laboratory mouse, and these differ from known genetic mechanisms that have been defined previously. Hence, the ability of some strain types to expand in the environment as a consequence of enhanced transmission may also lead to the spread of virulence determinants.

## Introduction

*Toxoplasma gondii* is a globally distributed protozoan parasite with a broad host range, and which often incidentally infects humans [1]. Studies of the population genetics have emphasized the predominance of three clonal lineages that share overlapping geographic distributions and hosts in North America and Europe [2]. In addition, a fourth clonal lineage, related to type II has recently been described in North America, where it tends to be found in wild rather than domestic animals [3]. In contrast, strains in South America are more genetically diverse and comprise distinct lineages that do not demonstrate marked clonality [2]. Recent studies, comparing more than 900 isolates from around the world, grouped strains into 15 separate haplogroups (HG) that cluster within 6 major clades [4]. Current coverage of isolates from Africa, Asia, and South East Asia is sparse, so this pattern is likely to change with continued sampling.

Infection with *T. gondii* normally causes only mild symptoms in healthy hosts [5], yet there is evidence that some strains, particularly in South America, are more virulent [6]. For example, in regions of southern Brazil, toxoplasmosis is associated with severe recurrent ocular disease [7] and strains from this region have been shown to comprise unique genotypes that are also highly virulent in mice [8], [9]. Previous studies have emphasized the genetic diversity of *T. gondii* strains collected in the jungles of French Guiana versus those collected in anthropized environments [10]. Infection of humans with strains from wild or jungle environments has been associated with severe toxoplasmosis in French Guiana [11], [12] [13], [14].

Although the factors that shape the population structure of *T. gondii* remain poorly understood, previous studies have highlighted the common inheritance of a monomorphic version of chromosome 1a (Chr1a) among clonal strains in North America and Europe [15]. It was originally proposed that association of monomorphic Chr1a with the dominant lineages in North America and Europe might explain their clonality, in particular if it were responsible for asexual transmission [15], [16]. However, subsequent studies found the identical version of Chr1a in several different lineages in South America, despite the fact that they are not clonal and differ substantially from each other across most other regions of their genomes [17]. The presence of monomorphic Chr1a among the clonal lineages might simply reflect their common recent ancestry [16], which resulted from a few genetic crosses in the wild [18]. However, such a process would not explain the presence of this same conserved chromosomal variant in otherwise disparate genomes of different halpogroups in South America. Rather, the presence of monomorphic Chr1a among lineages that differ substantially in their overall genomic content suggests it is preferentially retained despite recombination in other regions of the genome [17]. The reasons for the successful spread of Chr1a among such diverse strains from different geographic regions and within different genetic backgrounds remain unknown.

Here we examined the distribution of Chr1a in wild and anthropized *T. gondii* strains collected from French Guiana. We found the same monomorphic version of Chr1a was almost universally found in strains from anthropized environments, while this chromosome was extremely diverse in wild isolates. We were interested in factors that might lead to successful expansion of strains harboring Chr1a and considered both the potential of such isolates to cause acute and chronic infection in rodents and to be transmitted by cats. Several of the isolates from the anthropized environment were shown to have enhanced virulence in mice, despite not containing previously identified virulence factors, pointing to the existence of unique pathogenicity determinants in natural isolates of *T. gondii*. As well, strains harboring the monomorphic Chr1a showed a greater propensity for transmission in domestic cats. Together these factors may lead to greater success of strains in natural hosts within anthropized environments, which may in turn affect the spread of pathogenecity determinants important in humans.

## Methods

### Ethics statement

All animal experiments were conducted according to the U.S.A. Public Health Service Policy on Humane Care and Use of Laboratory Animals (Animal Welfare Assurance numbers A-3381-01 or A4400-01). Animals were maintained in an Association for Assessment and Accreditation of Laboratory Animal Care International-approved facilities. All protocols were approved by the Institutional Care Committee at the School of Medicine, Washington University in St. Louis (approval number 20130130) or the Animal Parasitic Disease Laboratory (protocol numbers 12-016 and 12-013), USDA, Beltsville, MD.

### Growth of *T. gondii* strains

Eighteen strains of *T. gondii* from French Guiana and one from Surinam were collected and characterized with microsatellite markers, described previously [10], [13], [19], [20]. These strains are designated with a code according to the *Toxoplasma* Biological Resource Center (BRC) (http://www.toxocrb.com) nomenclature for *T. gondii* isolates (Table S1). Thirty eight representative strains for the major HGs of *T. gondii* were chosen from a previously described set of strains [4] (Table S1). Strains were grown in human foreskin fibroblast (HFF) cells cultured in DMEM (Invitrogen) containing 10% FBS, 2 mM glutamine, 20 mM HEPES pH 7.5 and 10 µg/ml gentamicin, and harvested after host cell lysis by passing through 3.0 micron polycarbonate filters (Fisher Scientific, UK) [4]. Harvested parasites were resuspended in phosphate buffered saline (PBS) at a concentration of approximately 10^6^ cell/ml and digested with 10 µg/ml proteinase K (Sigma, St. Louis, MO, USA) at 55°C for 2 hr. Proteinase K was inactivated by incubating the lysate in 95°C for 15 min [4].

### Genotyping with microsatellite markers

*T. gondii* strains were genotyped using 15 microsatellite markers distributed on 10 of 14 chromosomes, as described previously [21]. Briefly, for each primer pair, the forward primer was 5′-end labeled with fluorescein to allow sizing of PCR products that were separated by electrophoresis in an automatic sequencer. PCR was carried out in a 25 µL reaction mixture consisting of 12.5 µL of 2X QIAGEN Multiplex PCR Master Mix (Qiagen, France), 5 pmol of each primer, and 5 µL of template DNA. Cycling conditions were 15 min at 95°C; 30 s at 94°C, 3 min at 61°C, and 30 s at 72°C for 35 cycles followed by 30 min at 60°C. PCR products were diluted 1∶10 with deionized formamide. One microliter of each diluted PCR product was mixed with 0.5 µL of a dye-labeled size standard ROX 500 (Applied Biosystems, location) and 23.5 µL of de-ionized formamide (Applied Biosystems). The mixture was denatured at 95°C for 5 min and then electrophoresed using an ABI PRISM 3130xl automatic sequencer (Applied Biosystems). The sizes of the alleles in bp were estimated using GeneMapper analysis software v4.0 (Applied Biosystems).

Neighbor-Joining trees were reconstructed from the genetic distances among individual isolates using Populations 1.2.32 (http://bioinformatics.org/populations/). Trees were reconstructed using the Cavalli- Sforza and Edwards chord-distance estimator [22]. Distance analyses were repeated for 1,000 bootstrap replicates in which loci were sampled with replacement. Unrooted trees were generated using MEGA 6.05 (http://www.megasoftware.net/history.php) software.

### Genotyping of introns, ChrIa regions, and RFLP markers

Genotyping of *T. gondii* strains was conducted using nine PCR-RFLP markers and sequencing of four introns from three different genes (*UPRT*, *EF*, and *HP*) constituting 1,775 bp. These markers have been used previously to characterize other *T. gondii* lineages [4], and representative strains from each HG were included here for comparison (Table S1). Genetic profiling of ChrIa was performed by sequencing 12 scattered blocks comprising 800 to 900 bp each for a total length 8,055 bp, as described previously [17], [23]. Parasite lysates were used as template DNA for the PCR amplification of RFLP markers, introns, and ChrIa regions using primers described previously [4]. Amplified PCR products were used for restriction enzyme digestion and gel electrophoresis or for sequencing using BigDye cycle sequencing (Applied Biosystems, Foster City, CA) performed by GENEWIZ, Inc. (South Plainfield, NJ). Clustal W/X [24] was used to align raw sequences with default settings. Aligned sequences were saved as nexus files and directly incorporated into SplitsTree v4.4 [25] to generate unrooted phylogenetic networks using a neighbor-net method and 1,000 bootstrap replicates.

### Acute infection in mice

Virulence was determined by intraperitoneal (i.p.) infection of eight week old female CD-1 outbred mice (Charles River Laboratories, Wilmington, MA) using 5 mice for doses of 10, 100, or 1000 tachyzoites (n = 15 mice per strain), as described previously [26]. In parallel, plaque assays were conducted to estimate the number of viable parasites, as described previously [26]. Animals were monitored for 30 days and surviving animals were tested serologically by enzyme-linked immunosorbent assay (ELISA). In brief, tachyzoites of the ME49 strain were sonicated and used to coat high-binding capacity ELISA plates (Corning, location) with a suspension containing 1×10^6^ parasites/ml in PBS at 4°C for overnight. Plates were washed using PBS containing Tween-20 (0.05%). Sera were diluted 1∶500 in 1.0% bovine serum albumin (BSA) in PBS and incubated with coated plates for one hour at room temperature. After incubation, plates were washed three times using PBS with Tween-20 (0.05%) and incubated for one hour at room temperature with horseradish peroxidase-conjugated goat anti-mouse IgG (Amersham Pharmacia/GE Healthcare, USA) diluted 1∶10,000 in PBS. Following washing, peroxidase reactivity was detected with 100 µl of equal mix of substrate-A and B (ECL, PerkinElmer, Waltham, MA, USA) for 20 min in the dark. The reaction was stopped using 50 µl of 2 M H_2_SO_4_ and the plates were scanned for absorbance at 490 nm. Samples were performed in triplicate wells and repeated twice. Mean values for each sample were compared to a set of 6 non-infected animals (negative controls) to establish significance levels for positivity (95%), as described previously [27]. Cumulative mortality was defined as  =  (the number of animals that succumbed/total number of animals that were infected) ×100.

### Chronic infection in mice and transmission studies

For strains used to study transmission dynamics, we specifically chose strains with a low passage history, all within 5 passages of primary isolation. To develop tissue cysts for transmission studies, CD1 mice were infected by i.p. injection of tachyzoites of *T. gondii* strains grown in culture. For highly virulent strains, CD1 mice were treated with sulfadiazine (0.4 to 0.6 mg/ml) supplemented drinking water for 10 days (5 to 15 days post-infection). At 6 to 8 weeks for post-infection, mice were euthanized and brains were removed and homogenized in sterile PBS. The number of tissue cysts was determined by fluorescent staining with fluorescein isothiocyanate-conjugated *Dolichos Biflorus* lectin (DBL) and microscopic counting. In brief, an aliquot of the homogenized brain was fixed in 5% formalin and permeabilized with 0.25% Triton X-100. Samples were washed three times by centrifugation (400 *g* for 10 min) at room temperature in PBS, followed by blocking in 10% FBS for 20 min and staining with DBL (Sigma, St, Louis, MO). Samples were examined and cysts counted using a wide-field epifluorescence microscope. Groups of 5 CD1 mice were used for oral challenge with ∼20 cysts each, as described previously [8]. At 30 days post-infection, sera were collected from surviving mice, and tested by ELISA, as described above. Oral transmission was defined as the number of animals that were infected (i.e. those that became sick and died plus those that were serologically positive), over the total number of animals challenged ×100, as defined previously [8].

To monitor transmission in the definitive host, *Toxoplasma*-free laboratory raised cats were fed orally with brain homogenates containing tissue cysts of *T. gondii* (100–800 cysts per animal) and feces were examined for shedding of oocysts between days 4–21, as described previously [28]. Cats used in these experiments were aged 2–4 mos and of either sex and were part of a colony maintained at the USDA, as described previously [28]. For practical reasons, the experiments were staggered to assure similar age of the cats, and similar duration of chronic *T. gondii* infection in mice prior to feeding (1–2 mos after establishment of infection). Serological conversion of the inoculated cats was determined by the modified agglutination test as described previously [29]. For select cats, infections were tested by bioassay in Swiss Webster mice. Samples of heart, liver, lung, and spleen (total 50 g) were homogenized in saline, digested in 0.5% trypsin or pepsin, and inoculated s.c. into groups of 5 mice. Six weeks later mice were bled, tested serologically and their brains were examined for tissue cysts, as described above. For cats that were positive for shedding, the total number of oocysts shed was determined by pooling positive stool samples, making dilutions, and counting oocysts by microscopic examination. To monitor viability, oocysts were washed in HBSS and treated with 10% (vol/vol) Chrolox (8.25% NaOCl) for 30 min at 4°C. After further washing, oocysts were vortexed in a suspension of 0.3–0.5 g of 0.5 mm glass beads in 1 ml of PBS to mechanically disrupt the oocyst wall. Oocysts were further treated with 5% sodium taurodeoxycholate (Sigma-Aldrich) in HBSS for 10 min at 37C. Following washing, oocysts were plated on HFF monolayers and the number of plaques formed determined at 10 days post-infection.

### Statistics

Comparison of the frequency of oocyst shedding in cats was analyzed using Fisher's exact test as computed using Prism (GraphPad). To determine the minimum sample sizes needed to detect a significant difference at *P*<0.05, we used Pearson's Chi-squared test with continuity correction and assumed the same effect size as is evident in the current data (.59). Estimates were calculated with 1 degree of freedom under a chi-square test using R as described (http://www.statmethods.net/stats/power.html). Power analysis was computed using pwr: Basic functions for power analysis. R package version 1.1.1. (http://CRAN.R-project.org/package=pwr).

## Results

### Distribution of *T. gondii* strains isolated from French Guiana

**Figure 1:**
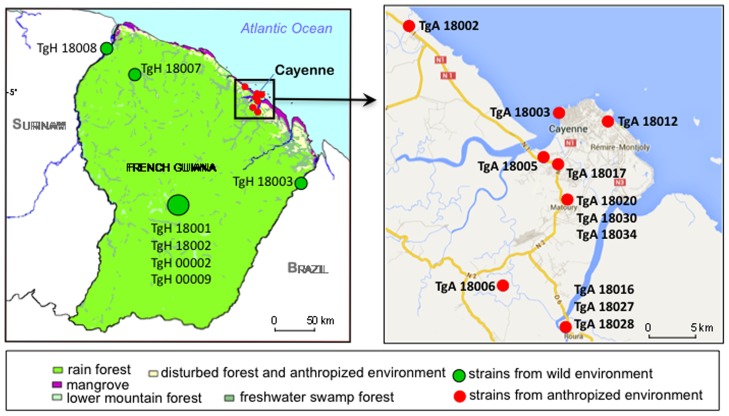
Distribution of strains from French Guiana. Map of French Guiana shows locations of strains collected from the wild Neotropical rainforest (7 strains, green points) vs. anthropized environment (12 strains, red points). Of the 7 wild strains, only 3 (TgH18003, TgH18007, and TgH18008) were mapped with precision in the rainforest. Because information is lacking for the remaining 4 wild strains in the Neotropical rainforest (TgH18001, TgH18002, TgH00002, and TgH00009), they are shown as a large green point in the center of the rainforest area. For strains from the anthropized environment, each point corresponds to the localization of one or more isolates sharing the same geographic origin.

French Guiana consists of two main geographical environments: a wild environment characterized by the dense, near-inaccessible, and unpopulated Neotropical rainforest that covers 96% of the country, and an anthropized environment that lies along a thin coastal strip where the majority of the human population lives in towns, villages or at the edge of forests that have been cleared for farming (Figure 1). Seven strains from the rainforest environment (TgH18001, TgH18002, TgH18003, TgH18007, TgH18008, TgH00002, and TgH00009) were isolated from blood samples of patients with severe toxoplasmosis between 1991 and 2004 [13], [19], [20]. All these patients were otherwise healthy adults who were infected naturally in the rainforest. The locations of these wild strains are available only for three strains (TgH18003, TgH18007, and TgH18008), which are shown precisely, while the remaining wild strains are grouped in a single central location (Figure 1). Twelve strains were collected from animals in the anthropized environment of French Guiana in 2009 [10]: seven were from stray dogs (TgA18002, TgA18003, TgA18006, TgA18012, TgA18017, TgA18020 and TgA18030), three were from chickens (TgA18016, TgA18027, and TgA18028), one was from a stray cat (TgA18034), and one was from a greater grison (*Galictis vittata*) (TgA18005). The 12 strains from the anthropized environment were collected within a 30-km radius in the Cayenne area, the main city of French Guiana (Figure 1).

### Genotyping and phylogenetic analysis of *T. gondii* strains from French Guiana

**Figure 2:**
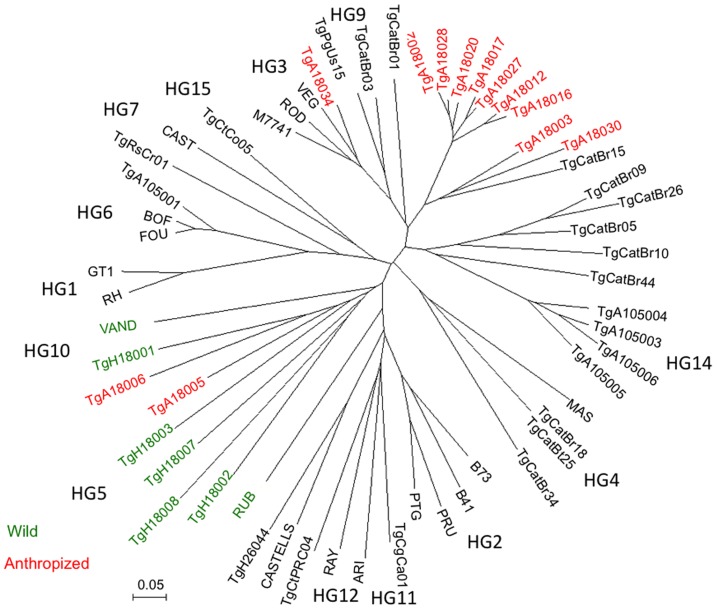
Analysis of *T. gondii* strains based on microsatellite markers. Strains isolated from anthropized environment (red) vs. wild Neotropical rainforest of French Guiana were compared to type strains representing the major haplogroups (HG). Neighbor-joining analysis of 15 microsatellite markers, displayed as an unrooted tree drawn using MEGA. Anthropized strains grouped with HG3 and HG9, while wild strains were deep branching and associated with a variety of divergent HGs. Color-coding indicates the region of origin: anthropized environment (red), wild Neotropical rainforest (green), HG reference strains (black).

Previous studies using microsatellite (MS) markers have emphasized that strains from the anthropized regions of French Guiana show lower allelic diversity than those form the wild, and that these two groups clustering separately using phylogenetic analysis [10]. To provide a broader comparison of these two groups of isolates from French Guiana, we compared them to representative strains from each of the major 15 HGs, recently defined by a combination of different genetic markers [4]. These reference strains were chosen to represent not only different HG, but also different geographic regions, and hosts (see Table S1). We analyzed the MS patterns for 57 strains, including 19 from French Guiana (7 wild, 12 anthropized) and clustered them using Neighbor-joining. As shown in Figure 2, the wild (green) and anthropized (red) isolates group to distinct branches of the unrooted tree together with various HGs. The anthropized strains lie on a common branch with HG3 and HG9, and yet they are slightly distinct from each of these groups (Figure 2). In contrast, the wild strains group with a variety of divergent HGs, including HG5 and HG10 that have previously been found in French Guiana and Brazil [4]. There were two exceptions to this grouping, wherein anthropized strains clustered with the wild strains (i.e. TgA18005, Tg18006) (Figure 2). Consistent with previous reports [10], anthropized strains show a high degree of similarity to each other, while the wild strains were found on separate long branches (Figure 2).

**Figure 3:**
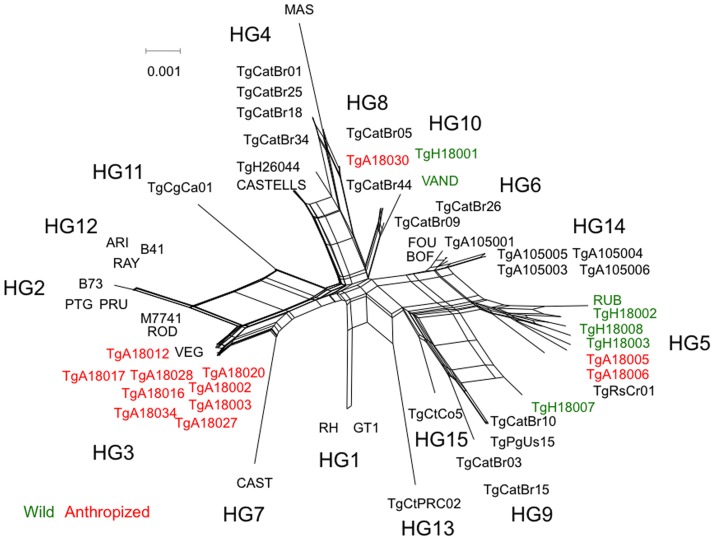
Analysis of *T. gondii* strains based on intron markers. Strains isolated from anthropized environment vs. wild Neotropical rainforest of French Guiana were compared to type strains representing the major haplogroups (HG designations). Neighbor-net analysis was conducted using four intron sequences (*UPRT*1, *UPRT*7, *EF*1, and *HP*2). Rainforest isolates clustered with two previously defined haplogroups 5 and 10, whereas anthropized isolates clustered with haplogroup 3. Color-coding indicates the region of origin: anthropized environment (red), wild Neotropical rainforest (green), and HG reference strains (black).

The dissimilarity of wild strains of French Guiana isolates might influence their grouping in the MS analysis due to a long-branch effect. Therefore, we also examined the genetic composition of French Guiana strains using a set of introns from housekeeping genes that have been used previously to analyze the population structure of *T. gondii* [4], [23]. This intron-based analysis provides an independent estimate of divergence based on single nucleotide polymorphisms (SNP) in selectively neutral loci. Analysis of genetic diversity based on intron polymorphisms using a neighbor-network revealed that anthropized strains were closely grouped with HG3, while wild strains grouped with HG5 or HG10. These groupings were supported by shared regions of the networks, and did not simply reflect long-branch attractions. As noted above for the microsatellite analysis, two strains from the anthropized environment (i.e. TgA18005, TgA18006) grouped with the wild strains in HG 5, and one strain (i.e. TgA18030) grouped with HG8 (Figure 3). Based on the relative branch lengths, anthropized strains also appeared much more tightly clustered, while wild strains were more divergent, consistent with the MS analysis above and reported previously [10].

**Figure 4:**
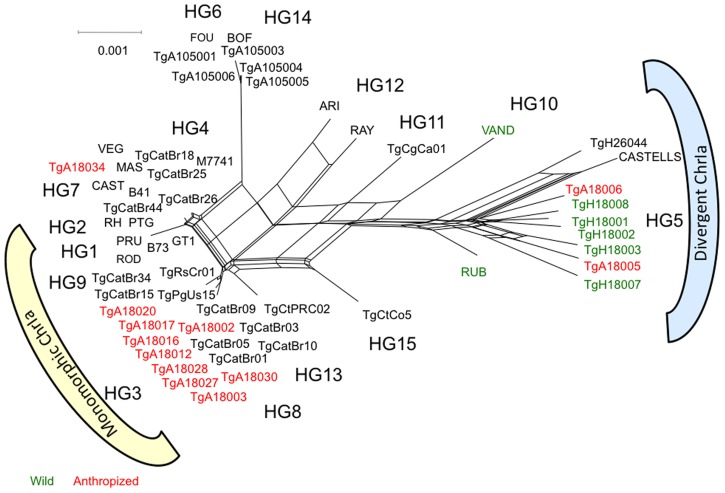
Neighbor-net analysis of genetic diversity on ChrIa. Neighbor net constructed using sequences from 12 reference blocks (∼800–900 bp each) of ChrIa from representative strains of *T. gondii*. A strong separation was evident between strains from anthropized and Neotropical rainforest environments of French Guiana. Isolates from anthropized environment harbored a monomorphic version of ChrIa, whereas rainforest isolates contained divergent forms of ChrIa. Color-coding indicates the region of origin and composition of ChrIa: anthropized environment (red), rainforest environment (green), HG reference strains (black), monomorphic ChrIa (yellow), and divergent ChrIa (blue).

The similarity of anthropized strains from French Guiana to HG3 suggested that these strains might share other traits common to this group; for example type III strains belong to HG3 and also harbor monomorphic Chr1a [15], [23]. To determine the composition of Chr1a in FG strains, we sequenced regions that were scattered across the chromosome and which have been used previously to characterize its genetic makeup [15], [23]. Analysis of SNPs within these regions by Neighbor-network analysis revealed that the majority of anthropized strains contained a monomorphic version of Chr1a, similar to HG1, 2, 3 (Figure 4). The exceptions to this pattern were strains TgA18006 and TgA18005, which as discussed below are wild-like strains collected from an anthropized environment. This monomorphic Chr1a is also found in HG4, 7, 8, 9, as reported previously [15], [23]. In contrast, the wild strains from French Guiana contained highly divergent versions of Chr1a, similar to other HG5 and HG10 strains (Figure 4).

The similarity of anthropized strains to HG3 and the presence of monomorphic Chr1a suggested they might represent a pocket of clonal type III strains in South American, despite the fact that this lineage is generally confined to North America and to a lesser extent Europe [4]. Hence, we analyzed a set of restriction fragment polymorphism (RFLP) markers that has previously been used in typing *T. gondii* strains [4]. Based on this analysis, the anthropized strains did not match type III strains (or HG3) but rather were mixtures of alleles from types I, II, and III (Table 1). This suggests that they may have arisen by natural recombination among the clonal lineages, or at least have a similar parental origin to the clonal types that dominate in North America and Europe. This mixed ancestry appears to have a predominant type III signature, consistent with the grouping by microsatellite, intron, and RFLP markers.

**Table 1:** Genotyping of French Guiana *T. gondii* strains using PCR-RFLP markers.

Strains	Genetic markers																										
	SAG1c			3′-SAG2^b^			BTUB			GRA6		C22-8			C29-2			PK1	Alt.SAG2^b^			L358			Apicoplast		
	*Hae*II	*Sau*96I	Genotype^c^	*Hin*fI	*aTaq*I	Genotype^c^	*Bsi*E1	*aTaq*I	Genotype^c^	*Mse*I	Genotype^c^	*BsmA*I	*Mbo*II	Genotype^c^	*Hpych*4IV	*Rsa*I	Genotype^c^	*Ava*I	*Hin*fI	*aTaq*I	Genotype^c^	*Hae*III	*Nia*III	Genotype^c^	*AfI*II	*Dde*I	Genotype^c^
GT1 (Type I)	I^a^	I	1	I/II	I/III	1	I	I/III	1	I	1	I	I/II	1	I/II	I/III	1	I/III	I/II	I/III	1	I	I/III	1	I/II	I/III	1
PTG (Type II)	II/III	II/III	2/3	I/II	II	2	II/III	II	2	II	2	II/III	I/II	2	I/II	II	2	II	I/II	II	2	II/III	II	2	I/II	II	2
CTG (Type III)	II/III	II/III	2/3	III	I/III	3	II/III	I/III	3	III	3	II/III	III	3	III	I/III	3	I/III	III	I/III	3	II/III	I/III	3	III	I/III	3
TgA18020 (A)^e^	I	I	1	III	I/III	3	I	I/III	1	III	3	II/III	I/II	2	III	I/III	3	I/III	III	I/III	3	II/III	I/III	3	III	I/III	3
TgA18028 (A)	I	I	1	III	I/III	3	I	I/III	1	III	3	II/III	I/II	2	III	I/III	3	I/III	u1^d^	I/III	u1^d^	II/III	I/III	3	III	I/III	3
TgA18012 (A)	I	I	1	I/II	I/III	1	I	I/III	1	III	3	II/III	I/II	2	III	I/III	3	I/III	I/II	I/III	1	II/III	I/III	3	III	I/III	3
TgA18003 (A)	I	I	1	III	I/III	3	I	I/III	1	III	3	II/III	I/II	2	III	I/III	3	I/III	III	I/III	3	II/III	I/III	3	III	I/III	3
TgA18006 (W)	I	I	1	I/II	II	2	II/III	I/III	3	III	3	I	III	u1^d^	III	I/III	3	I/III	I/II	I/III	1	I	u1^d^	u1^d^	I/II	I/III	1
TgH18007 (W)	I	I	1	I/II	II	2	II/III	I/III	3	III	3	I	I/II	1	III	I/III	3	I/III	I/II	I/III	1	I	I/III	1	III	I/III	3
TgH18008 (W)	I	I	1	I/II	II	2	II/III	I/III	3	III	3	I	I/II	1	u1^d^	u1^d^	u1^d^	I/III	I/II	I/III	1	I	u1^d^	u1^d^	III	I/III	3

a Allele types are based on patterns seen in clonal type I, II and III strains.

b The marker *3′-SAG2* and *Alt.SAG2* target different DNA fragments in the *SAG2* gene.

c Genotype based on combined genetic profile characterized by the digestion pattern using both enzymes.

d designates an unique allele.

e Anthropized (A) vs. wild (W) strains.

### Acute virulence of *T. gondii* strains from French Guiana

The clonal lineages type I, II and III (HGs 1, 2, and 3) differ substantially in their acute virulence in mice, with type I strains being acutely virulent, type II strains exhibiting intermediate virulence, and type III being avirulent in laboratory mice [2]. Previously genetic studies have shown that these phenotypic differences are largely due to the combination of alleles at a few polymorphic loci that encode rhoptry kinases or pseudokinases [30]. We examined the ability of a subset of the anthropized and wild strains from French Guiana to infect outbred laboratory mice and cause acute infection. The wild strains from French Guiana exhibited high levels of acute virulence (Table 2), consistent with previous reports that they may cause more severe disease [19]. In contrast, challenge with anthropized strains resulted in a wide range of mortality from 0–100% for different strains (Table 2). The high degree of lethality seen in some anthropized strains (i.e. TgA18020) is not consistent with type III strains, which normally do not result in any mortality at even much higher doses [26]. The mixed genotypes of these anthropized strains (Table 1), suggests that their more virulent phenotypes may be a consequence of their recombinant genotypes.

**Table 2:** Virulence and transmission of *T. gondii* strains isolated from French Guiana.

Strain	Host	Chr1a	Virulence^a^ (%)	Oral Transmission (%)^b^	Cat infection^c^		Genotyping			
					Oocysts	Serum^d^	*ROP5*	*ROP18*	*ROP16*	*GRA15*
TgA18020	Dog	Monomorphic	100	100	No shedding	<5^g^	I	III	I/III	I/III
					1.8×10^7^	640				
TgA18028	Chicken	Monomorphic	33	100	1.5×10^8^	sc	I	III	I/III	I/III
					2.7×10^8^	320				
TgA18012	Dog	Monomorphic	15	100	No shedding	160^g^	I	III	I/III	I/III
					1.0×10^8^	>3,200				
TgA18003	Dog	Monomorphic	0	100	1.3×10^8^	sc	I	III	I/III	I/III
					1×10^6^	640				
TgA18006	Dog	Divergent	73	80	No shedding	100	u1^e^	I^f^	I/III	I/III
					No shedding	<25				
TgH18007	Human	Divergent	90	80	2×10^8^	200	u1^e^	I^f^	I/III	I/III
					No shedding	<5				
TgH18008	Human	Divergent	83	100	No shedding	<5	u1^e^	I^f^	I/III	I/III
					No shedding	40				

a Cumulative mortality of CD1 mice infected intraperitoneally with 10, 100, and 1000 parasites/mouse (5 mice/dose, 15 mice/strain).

b Oral transmission using CD1 mice infected orally with 20 tissue cysts/mice (5 mice/strain).

C Challenge of cats with 100–800 tissue cysts/cat.

d Titer, generally considered positive when ≥25.

e Unique genotype other than type I, II, and III.

f related to type I allele.

g negative by bioassay in mice.

nd, not done.

Sacrificed at day 10 prior to development of positive titer.

To evaluate if the outcome of mouse infections was related to previously characterized virulence traits, we analyzed polymorphism in *ROP18*, *ROP5, ROP16* and GRA15. All of the anthropized stains contained a type III allele at *ROP18*, which has previously been associated with the avirulence of type III strains [26], [31]. The anthropized strains also contained the shared alleles characteristic of type I and III strains at *ROP5*, *ROP16* and *GRA15* (Table 2). Hence, the range of virulence phenotypes of anthropized strains cannot be explained by the known virulence determinants of *T. gondii*. The wild strains from French Guiana contained divergent alleles at both *ROP5* and *ROP18* (Table 2), indicating they contain additional allelic diversity not previously seen in the clonal lineages.

### Chronic infection and transmission of *T. gondii* strains from French Guiana

We also tested the ability of strains from French Guiana to cause chronic infection in mice and to generate tissue cysts that were orally infectious to mice and to cats. For strains that exhibited high virulence, mice were treated with sulfadiazine to prevent death. All of the strains produced normal appearing tissue cysts in the brains of chronically infected animals and these were orally infectious to naïve mice (Table 2). One potential model to explain the preponderance of Chr1a in the environment would be if it favors infection of cats or leads to greater fecundity (i.e. higher oocyst production) in cats. To test this possibility, the brains from chronically infected mice were fed to each of two cats per parasite strain. Feeding of tissue cysts from all four anthropized strains harboring the monomorphic Chr1a resulted in oocyst shedding by cats, although for two strains only one of the two cats challenged were positive (in total 6 of 8 cats challenged shed oocysts) (Table 2). The two animals that did not shed oocysts also did not become infected as shown by bioassay (Table 2). In contrast, only one of three divergent strains, harboring divergent forms of Chr1a, resulted in shedding of oocysts when fed to cats, and this only occurred in one of two cats challenged, while the remaining animal did not become infected as shown by its low serum titer (Table 2). For two of the divergent strains (TgA18006, TgH18008), one of two cats fed tissue cysts for each strain likely became infected as shown by a positive antibody titer, yet none of the four cats shed oocysts (Table 2). Overall, 5 of 6 cats fed tissue cysts from these divergent strains failed to shed oocysts (Table 2). Among cats that shed, the number of oocysts and viability were similar (Table 2 and data not shown).

The low level of infectivity of strains harboring a divergent Chr1a is unexpected, as cats are normally quite permissive for infection when fed tissue cysts [32]. Therefore, we tested the null hypothesis that presence of the monomorphic Chr1a leads to greater transmission in cats using Fisher's exact test, which nearly achieved statistical significance (*P* = 0.0513, 1-tailed test), suggesting the observed outcome is unlikely to be due to chance. These findings support the hypothesis that anthropized strains containing a monomoprhic versio of Chr1a have a high capacity for productive infections in cats, while this trait is more restricted in wild strains harboring divergent forms of Chr1a.

## Discussion

Our findings reveal that *T. gondii* strains in French Guiana comprise two very different populations that are stably maintained despite their close geographic proximity. Highly divergent strains are typical of the jungle environment, while less diverse strains predominate in anthropized environments, with few exceptions. Consistent with previous reports, these anthropized strains are related to clonal type III, and are similar to other strains seen in the Caribbean [10]. However, they are not simply type III strains seen in North America, but instead have genotypes consisting of mixtures of alleles. These strains exhibited a range of acute virulence in the mouse, which cannot be explained by previous virulence determinants. Anthropized strains also share a monomorphic version of Chr1a, while wild strains are highly divergent, and this feature correlated with efficient oocyst shedding in cats, suggesting this chromosome may be important in transmission.

The majority of strains isolated in the anthropized environment were similar to HG3 and HG9, two related lineages that predominate in North America and South America, respectively [4]. Anthropized French Guiana strains have previously been analyzed using microsatellite makers and were shown to be similar to type III-related genotypes that are common in the Caribbean [10]. Our studies using a wider range of markers demonstrate that they are not simply variants of type III, but rather contain alleles from different genetic types at different loci. This pattern suggests that they may be derived from genetic crosses in the wild between the members of the clonal lineages, or between a unique parental strain and a type III strain. Further analysis of their genetic composition may reveal their ancestry and relationship to existing HG. Although these anthropized strains differ from clonal type III strains, they have a highly conserved version of Chr1a that has previously been seen in both clonal North American strains and in non-clonal, genetically divergent South American strains [17], [23]. The mixed ancestry of French Guiana anthropized strains suggests they may have acquired Chr1a by interbreeding, perhaps from a type III-like parental donor.

There were several exceptions to the pattern that anthropized strains in French Guiana resemble HG3 and HG9. Notably, sample TgA18030 is more typical of HG4 and HG8, which are common in other regions of South America such as Brazil [4]. Additionally, two strains collected in the anthropized environment group much more closely with the wild strains (i.e TgA18006, TgA18005), both in the diversity of their genome as a whole and in having a divergent Chr1a. For this reason, they are denoted as wild (W) strains here. These strains were isolated from a dog (TgA18006) and a greater grison (TgA18005), in different geographic regions (Figure 1). The grison is a mustelid that has an omnivorous diet and typically inhabits aquatic regions of tropical savannahs, but is also known to enter areas that have active farming. The animal sampled here was in an anthropized environment, but may well have acquired its infection from the nearby jungle. Likewise, the close proximity of the jungle environment to developed areas of French Guiana makes it possible that the dog carrying TgA18006 was infected in the wild environment, despite being sampled in an anthropized region. These two examples also demonstrate that mixing of genotypes is likely to occur along this interface, something that has been suggested previously [10]. As such, attributes found in strains from the wild or anthropized environments, including virulence determinants, may spread from one population to another.

Isolates from wild regions of French Guiana were acutely virulent in the outbred mouse model, similar to strains from the related HG5 and HG10 [8]. The wild strains studied here contain divergent alleles at *ROP5* an *ROP18*, suggesting there may be important functional differences conveyed by these new alleles. The anthropized strains exhibited a wide range of virulence in the mouse model consistent with their mixed genotypes and distinct from what is expected from type III strains [26]. The intermediate to high virulence phenotype of some anthropized strains is similar to TgPgUs15 (also known as P89), a HG9 strain that is highly virulent in the mouse model despite having an allele of *ROP18* that is associated with avirulence [8], [17]. A similar pattern was seen here among anthropized strains that have a type III allele at *ROP18* and yet were highly virulent in naïve, outbred mice. *ROP18* encodes a serine threonine kinase that targets innate immunity and protects parasites within interferon-activated macrophages [33]. An upstream region in the genome of type III strains is associated with under-expression and avirulence [34]. Although the anthropized strains studied here contain type I alleles at *ROP5*, a cluster of pseudokinases that controls *ROP18* [35], [36], this is unlikely to explain their high level of virulence given the presence of a type III allele at *ROP18*. Collectively, these findings suggest that there are additional virulence traits in some anthropized strains such as those sampled here that cannot be easily explained by factors that have been identified in types I, II and III [30]. Further genetic studies will be necessary to define the genes, or combination of genes, that contribute to virulence in both the anthropized and wild strains from French Guiana, and other regions of South America.

Our studies reveal that the monomorphic Chr1a is more widespread than previously thought, being found in strains of mixed ancestry in anthropized regions of French Guiana. In each of the environments where the monomorphic Chr1a is prevalent, it is associated with anthropized environments. Clonal isolates (i.e. HG 1, 2, and 3) containing the monomorphic Chr1a in North America and Europe have been sampled from human infections, companion, or domestic animals in close proximity with urban or agricultural environments. In South America, monomorphic Chr1a is found in HG 4, 8, and 9 strains, most of which have been isolated from feral cats (*Felis catus*) and dogs and chickens in anthropized environments. A highly similar version of Chr1a also exists in China, where it is found in a predominant clonal lineage isolated from farming communities [4], [17]. In other regions, chimeric versions of Chr1a have been found in HG6 and HG14 strains, which are found in Africa and South America [4], [17]. As well chimeric versions of Chr1 have been seen in HG12, a newly described clonal lineage in North America [3], [4], [17]. Overall, the number of variants of Chr1a is small, and most lineages have inherited all or a large part of this chromosome from a single source. The exceptions are the highly divergent strains from lineages 5, 10, and 15 [4], [17], including the wild strains sampled here from French Guiana.

The wide spread distribution of the monomorphic Chr1a among genetically divergent lineages suggests several possible models for its unusual prevalence. It is possible that it was introduced into new environments by the spread of humans, together with domestication of a small number of animal species. Recent human mass migrations include the Neolithic agricultural revolution ca 10,000 years ago and widespread global colonization over the past 500 years. Travel between regions may have led to introduction of pest animals (i.e. mice and rats), domestic cats, or livestock, possibly carrying particular genotypes of *T. gondii* in the process. It has previously been noted that livestock serve as hosts for a number of parasites that show low genetic diversity [37], and this combined with human migrations, may serve to disseminate conserved strains of *T. gondii* across widely different geographic regions. However, this process has not been sufficient to erode the marked genetic divergence that is seen between North and South American strains of *T. gondii*. It also does not adequately explain the existence of the identical version of Chr1 in lineages that otherwise differ substantially in their genetic makeup and ancestry [17]. Rather this pattern suggests that once introduced into a lineage, Chr1 is advantageous. In North America, Chr1a is retained in inbreeding populations that show marked clonality. In contrast, in South America, it is retained in outbreeding populations that show high levels of diversity in their genome as a whole.

One potential trait that might influence the spread of strains bearing Chr1a would be if it conferred differences in fecundity or fertility in different felids, the only known definitive host [1]. Previous studies have shown that *T. gondii* strains often lose the ability to complete the life cycle within cats with extensive passages [38], hence we were careful to examine only low-passage isolates here. To test the possibility that Chr1a is an adaptation for transmission in domestic cats, we challenged cats with tissue cysts from either anthropized or wild strains from French Guiana and examined oocyst shedding. Anthropized strains that harbored the monomorphic Chr1a showed a high capacity for oocyst shedding, where 6 of 8 animals fed tissue cysts led to shedding of oocysts. This pattern is consistent with previously studied clonal lineages, which all share the monomorpic Chr1a [32], [39]. In contrast, only one of six cats fed *T. gondii* strains harboring divergent Chr1a shed oocysts, indicating they were less capable of causing productive infections in domestic cats. Although Chr1a is strongly associated with transmission in the domestic cat, not all monomorphic strains led to oocyst shedding, nor did all divergent ones fail to do so. Such variation suggests that although Chr1a may influence this trait, that other genes are likely also involved. None of the strains studied here are isogenic and so it is expected that complex phenotypes might not track completely with any one chromosome or gene. Additionally, although we have controlled for passage in the laboratory, these the strains likely differ in passage history in the wild. They were isolated from different hosts and it is possible that such variable histories might influence subsequent transmission in the domestic cat.

Although there was a strong association between the monomorphic Chr1a and cat transmission, it slightly exceeded the cutoff needed to achieve statistical significance by the normally accepted value of *P*<0.05. Such limitation might be overcome by larger sample sizes, but this is offset by ethical concerns about the use of more animals. To provide a power estimate, we performed Pearson's Chi-squared test with continuity, which indicated that ∼30 animals would be needed to have 90% power to reliably detect an effect of this size at *P*<0.05. Thus confirmation of the trend detected here would require almost doubling the number of animals used in the present study. Moreover, this would only test the association between the monomorphic Chr1a and cat transmission among this relatively small set of strains from French Guiana, but not establish if this pattern were true on a wider basis. At present, the only other example of a wild strain that showed low capacity to cause oocyst shedding when tissue cysts were fed to cats is an unrelated isolate from an Alaskan black bear (*Ursus americanus*) [40]. RFLP typing of this isolate indicates it has a mixed lineage and is not a member of one of the predominant clonal lineages in North America [40]; suggesting that it is a member of a divergent lineage, although the genotype of Chr1a in this strain is unknown. Our findings support the hypothesis that variation in Chr1a is associated with enhanced cat transmission, and justify more widespread testing of this pattern among other strains of *T. gondii*.

Domestic house cats are found in French Guiana among anthropized environments, although specific data on their densities, home range, etc., are not available. In contrast, jungle environments are unlikely to be populated by domestic cats but instead have wild felids including ocelot (*Leopardus pardalis*), oncilla (*Leopardus tigrinus*), Margay (*Leopardus wiedii*), cougar (*Puma concolor*), jaguarundi (*Puma yagouaroundi*) and jaguar (*Panthera onca*) [41]. Although *T. gondii* has been isolated from the tissues of a jaguar in French Guiana [20], the role of wild felids in natural transmission in the jungle is unknown. Hence, differences in transmission among felid species may be one factor that maintains the separation of wild and anthropized strains in French Guiana.

Wild and anthropized strains also showed similar abilities to cause chronic infection in laboratory mice, and they were equally capable of producing tissue cysts that were orally infectious to naive mice. However, strains of laboratory mice are extremely closely related [42], and they do not capture the genetic diversity of wild house mice from which they were derived. Hence, Chr1a may be adapted for transmission in natural rodent species such as house mice (*Mus musculus*), or species of rats (i.e. *Ratus norvegicus or Rattus rattus*) that commonly inhabit anthropized environments in many different geographic regions. In contrast, wild strains of *T. gondii* are likely transmitted by distinct species of rodents that populate jungle environments [43], [44]. Future studies designed to sample a range of intermediate hosts, as well as the species of felids involved in transmission, will be needed to address the hypothesis that Chr1a is an adaptation for transmission among distinct hosts in the wild. French Guiana is ideally suited for such sampling as it contains highly divergent and genetically homogenous lineages that exist in close proximity in a region of high transmission. Moreover, Chr1a is strongly associated with both genotype and environment along a well-defined boundary between anthropized and wild environments. Such studies on the ecology of natural transmission have the potential to inform us about the spread of *T. gondii* strains, and the virulence traits they carry, among natural hosts that lead to zoonotic infections.

## Supporting Information

Table S1 *Toxoplasma gondii* strains used for population genetic analysis.
(PDF)
