# Supplementary material for: Geographic Separation of Domestic and Wild Strains of Toxoplasma gondii in French Guiana Correlates with a Monomorphic Version of Chromosome1a
Source: PLoS Negl Trop Dis. 2014 Sep 18;8(9):e3182. doi: 10.1371/journal.pntd.0003182 (PMC4169257; doi:10.1371/journal.pntd.0003182)
Supplement: Table S1 — Toxoplasma gondii strains used for population genetic analysis. (PDF) [file pntd.0003182.s001.pdf]

Table S1. *Toxoplasma gondii* isolates used for population genetics analysis.

Isolate	Toxoplasma BRC code	Haplogroups	Geographical origin	Host	Microsatellite Markers															References
					TUB2	*W35*	*TgM-A*	*B18*	*B17*	*M33*	*MIV.1*	*MXI.1*	*M48*	*M102*	*N60*	*N82*	*AA*	*N61*	*N83*	
RH-88		1	America / Northern / USA	Human/ Adolescent	291	248	209	160	342	169	274	360	209	166	147	119	265	87	306	1
GT1	TgA00004	1	America / Northern / USA	Animal / Goat	291	248	209	160	342	169	274	358	209	168	145	119	265	87	306	1
B73		2	America / Northern / USA	Animal / Bear	289	242	207	160	336	169	274	356	213	190	142	111	267	101	310	1
PRU	TgH00001	2	Europe / Western / France	Human / Congenital	289	242	207	158	336	169	274	356	209	176	142	117	265	123	310	2
PTG	TgA00001	2	America / Northern / USA	Animal / Sheep	289	242	207	158	336	169	274	356	215	174	142	111	265	91	310	1
M7741		3	America / Northern / USA	Animal / Sheep	289	242	205	160	336	165	278	356	215	190	147	111	267	91	312	1
ROD-US		3	America / Northern / USA	Human / Transplant	289	242	205	160	336	165	278	356	213	190	147	111	267	89	314	1
VEG	TgH00005	3	America / Northern / USA	Human / AIDS	289	242	205	160	336	165	278	356	213	188	153	111	267	89	312	1
GUY-CAN-FAM-0001	TgA18002	3	America / Southern / French Guiana	Animal / Dog	291	242	205	162	342	165	278	356	213	164	142	109	265	87	312	3
GUY-CAN-FAM-0015	TgA18003	3	America / Southern / French Guiana	Animal / Dog	289	242	205	162	336	165	278	356	227	164	142	111	265	89	312	3
GUY-CAN-FAM-0016	TgA18012	3	America / Southern / French Guiana	Animal / Dog	291	242	205	162	342	165	278	356	213	164	142	109	277	89	312	3
GUY-GAL-DOM-0005	TgA18016	3	America / Southern / French Guiana	Animal / Chicken	291	242	205	162	342	165	278	356	213	164	142	109	277	85	312	3
GUY-CAN-FAM-0004	TgA18017	3	America / Southern / French Guiana	Animal / Dog	291	242	205	162	336	165	278	356	213	164	142	109	267	85	312	3
GUY-CAN FAM 0018	TgA18020	3	America / Southern / French Guiana	Animal / Dog	291	242	205	162	336	165	278	356	213	164	142	109	265	89	312	3
GUY-GAL-DOM-0006	TgA18027	3	America / Southern / French Guiana	Animal / Chicken	291	242	205	162	336	165	278	356	213	164	142	109	267	89	312	3
GUY-GAL-DOM-0007	TgA18028	3	America / Southern / French Guiana	Animal / Chicken	291	242	205	162	336	165	278	356	213	164	142	109	265	87	312	3
GUY-FEL-CAT-0009	TgA18034	3	America / Southern / French Guiana	Animal / Cat	289	242	205	160	336	165	278	356	213	190	145	111	269	89	312	3
MAS	TgH00006	4	Europe / Western / France (imported)	Human / Congenital	291	242	205	162	362	169	272	358	221	166	142	111	332	95	338	1
TgCatBr01		4	America / Southern / Brazil	Animal / Cat	289	242	205	160	342	165	278	358	233	164	147	111	316	89	308	4
TgCatBr18		4	America / Southern / Brazil	Animal / Cat	291	242	207	160	338	169	272	358	229	164	142	111	263	89	308	4
TgCatBr25		4	America / Southern / Brazil	Animal / Cat	291	242	207	160	338	169	272	358	229	164	142	111	263	89	308	4
TgCatBr34		4	America / Southern / Brazil	Animal / Cat	291	248	205	160	338	169	272	356	245	164	136	111	316	87	314	4
GUY-KOE	TgH18002	5	America / Southern / French Guiana	Human / Amazonian toxoplasmosis	289	246	203	160	337	165	274	356	209	172	136	111	251	109	310	5
GUY-MAT	TgH18003	5	America / Southern / French Guiana	Human / Amazonian toxoplasmosis	291	242	203	160	339	165	272	358	221	174	138	107	277	95	312	5
GUY-RUB	TgH00002	5	America / Southern / French Guiana	Human / Amazonian toxoplasmosis	289	242	205	170	360	167	274	356	223	190	142	109	259	85	312	5
TgRsCr01		5	America / Central / Costa Rica	Animal / Toucan	291	248	205	160	364	165	274	356	209	192	140	115	263	97	304	6
GUY-GAL-VIT-0001	TgA18005	5	America / Southern / French Guiana	Animal / Greater grison	289	242	203	160	336	165	272	356	213	176	140	105	279	87	312	3
GUY-CAN-FAM-0007	TgA18006	5	America / Southern / French Guiana	Animal / Dog	289	246	209	160	342	173	272	356	239	168	138	113	261	87	312	3
GUY-MEL	TgH18007	5	America / Southern / French Guiana	Human / Amazonian toxoplasmosis	289	242	203	158	344	171	272	356	209	182	149	121	265	89	317	7
GUY-ABE	TgH18008	5	America / Southern / Suriname	Human / Amazonian toxoplasmosis	289	246	203	158	338	167	276	354	213	168	138	111	281	93	318	8
FOU	TgH00007	6	Europe / Western / France (imported)	Human / Transplant	291	248	205	160	342	165	274	354	227	166	147	111	281	89	306	1
BOF		6	Europe / Western / Belgium (imported)	Human / AIDS	291	248	205	160	342	165	274	354	227	166	147	111	273	89	306	1
TgCatBr09	TgA00008	6	America / Southern / Brazil	Animal / Cat	291	242	205	160	362	165	278	354	227	174	140	111	269	89	308	4
TgCatBr26		6	America / Southern / Brazil	Animal / Cat	291	248	205	160	362	165	278	354	229	174	140	111	271	89	308	4
GAB3-2007-GAL-DOM2	TgA105001	6	Africa / Central / Gabon	Animal / Chicken	291	248	205	160	342	165	274	354	223	166	147	111	269	89	306	9
CAST	TgH00008	7	America / Northern / USA	Human / AIDS	291	242	205	158	342	167	276	356	211	168	147	119	279	87	306	1
TgCatBr05	TgA00007	8	America / Southern / Brazil	Animal / Cat	291	242	205	160	362	165	278	356	237	174	140	111	265	89	314	4
TgPgUs15 (P89)	TgA00005	9	America / Northern / USA	Animal / Pig	291	242	205	160	348	165	278	356	213	190	142	111	261	87	314	1
TgCatBr3		9	America / Southern / Brazil	Animal / Cat	289	242	205	160	348	165	278	356	213	190	142	111	263	113	312	4
TgCatBr10		9	America / Southern / Brazil	Animal / Cat	291	242	207	160	360	165	278	356	229	174	140	105	263	91	314	4
TgCatBr15		9	America / Southern / Brazil	Animal / Cat	289	242	205	162	344	165	278	358	225	164	142	111	263	105	312	4
GUY-DOS	TgH18001	10	America / Southern / French Guiana	Human / Amazonian toxoplasmosis	289	246	203	160	344	167	272	356	229	176	142	113	263	85	312	5
GUY-VAND	TgH00009	10	America / Southern / French Guiana	Human / Amazonian toxoplasmosis	291	242	203	162	344	167	276	356	217	170	142	113	277	91	308	5
GUY-CAN-FAM-0019	TgA18030	10	America / Southern / French Guiana	Animal / Dog	289	242	205	162	336	165	278	356	219	164	145	105	273	93	312	3
TgCatBr44		10	America / Southern / Brazil	Animal / Cat	291	242	205	162	342	165	278	356	231	166	153	111	265	91	344	4
TgCgCa01		11	America / Northern / Canada	Animal / Cougar	289	242	205	158	336	169	274	354	219	174	151	119	259	79	332	10
ARI		12	America / Northern / USA	Human / Transplant	289	242	209	158	336	169	274	362	215	170	147	131	295	89	316	1
B41		12	America / Northern / USA	Animal / Bear	289	242	207	162	336	169	274	356	213	170	142	111	287	107	316	1
RAY		12	America / Northern / USA	Human / Congenital	289	242	211	160	336	169	274	362	233	176	151	113	283	99	320	1
TgCtPRC04[a]		13	Asia / Eastern / China	Animal / Cat	293	242	211	160	336	169	274	354	215	172	145	123	281	93	308	11
GAB2-2007-GAL-DOM2	TgA105004	14	Africa / Central / Gabon	Animal / Chicken	291	242	207	160	342	165	278	354	223	166	142	111	277	97	310	9
GAB3-2007-GAL-DOM9	TgA105005	14	Africa / Central / Gabon	Animal / Chicken	291	242	207	160	342	165	278	354	229	166	142	111	273	95	310	9
GAB1-2007-GAL-DOM10	TgA105003	14	Africa / Central / Gabon	Animal / Chicken	291	242	207	160	342	165	278	354	225	166	142	111	275	97	310	9
GAB5-2007-GAL-DOM6	TgA105006	14	Africa / Central / Gabon	Animal / Chicken	291	242	207	160	342	165	278	354	225	166	145	111	275	101	310	9
TgCtCo05		15	America / Southern / Colombia	Animal / Cat	291	242	205	160	336	165	276	356	223	166	142	121	279	87	304	12
CASTELLS		4	America / Southern / Uruguay	Animal / Sheep	287	242	207	158	358	169	274	356	239	164	138	109	283	87	324	1
TgH26044	TgH26044	4	Europe / Western / France	Human / Congenital	287	242	207	158	354	169	274	356	221	168	155	109	281	103	322	This study

[a] TgCtPRC02 is a related strain with a similar genotype (11).

References:

1. Howe DH and Sibley LD. 1995. *Toxoplasma gondii* comprises three clonal lineages: correlation of parasite genotype with human disease. Journal of Infectious Diseases. 172:1561-1566.
2. Ajzenberg D, Bañuls AL, Tibayrenc M, Darde ML. 2002. Microsatellite analysis of *Toxoplasma gondii* shows considerable polymorphism structured into two main clonal groups. International Journal for Parasitology. 32:27-38.
3. Mercier A, Ajzenberg D, Devillard S, Demar MP, de Thoisy B, Bonnabau H, Collinet F, Boukhari R, Blanchet D, Simon S, Carme B, Dardé ML. Human impact on genetic diversity of Toxoplasma gondii: example of the anthropized environment from French Guiana. Infect Genet Evol. 2011 Aug;11(6):1378-87.
4. Pena HFJ, Gennari SM, Dubey JP, Su C. Population structure and mouse-virulence of *Toxoplasma gondii* in Brazil. International Journal for Parasitology. 2008. 38:561-569.
5. Carme B, Bissuel F, Ajzenberg D, Bouyne R, Aznar C, Demar M, Bichat S, Louvel D, Bourbigot AM, Peneau C, Neron P, Dardé ML. Severe acquired toxoplasmosis in immunocompetent adult patients in French Guiana. J Clin Microbiol. 2002 Nov;40(11):4037-44.
6. Dubey JP, Velmurugan G, Morales JA, Arguedas R, Su C. 2009. Isolation of *Toxoplasma gondii* from the keel-billed toucan (*Ramphastos sulfuratus*) from Costa Rica. Journal of Parasitology. 95:467-468.
7. Ajzenberg D, Bañuls AL, Su C, Dumètre A, Demar M, Carme B and Dardé ML. 2004. Genetic diversity, clonality and sexuality in *Toxoplasma gondii*. International Journal for Parasitology. 34: 1185-1196.
8. Demar M, Ajzenberg D, Maubon D, Djossou F, Panchoe D, Punwasi W, Valery N, Peneau C, Daigre JL, Aznar C, Cottrelle B, Terzan L, Dardé ML, Carme B. Fatal outbreak of human toxoplasmosis along the Maroni River: epidemiological, clinical, and parasitological aspects. Clin Infect Dis. 2007 Oct 1;45(7):e88-95.
9. Mercier A, Devillard S, Ngoubangoye B, Bonnabau H, Bañuls AL, Durand P, Salle B, Ajzenberg D, Dardé ML. 2010. Additional haplogroups of Toxoplasma gondii out of Africa: population structure and mouse-virulence of strains from Gabon. PLoS Neglected Tropical Diseases. 4: e876.
10. Dubey JP, Quirk T, Pitt JA, Sundar N, Velmurugan GV, Kwok OCH, Leclair D, Hill R, and Su C. 2008. Isolation and Genetic Characterization of *Toxoplasma gondii* from Raccoons (*Procyon lotor*), Cats (*Felis domesticus*), Striped Skunk (*Mephitis mephitis*), Black Bear (*Ursus americanus*), And Cougar (*Puma concolor*) from Canada. Journal of Parasitology. 94:42-45.
11. Zhou P, Zhang H, Lin RQ, Zhang DL, Song HQ, Su C, Zhu XQ. Genetic characterization of Toxoplasma gondii isolates from China.Parasitol Int. 2009 Jun;58(2):193-5.
12. Dubey JP, Su C, Cortes JA, Sundar N, Gomez-Marin JE, Polo LJ, Zambrano L, Mora LE, Lora F, Jimenez J, Kwok OC, Shen SK, Zhang X, Nieto A, Thulliez P. 2006. Prevalence of *Toxoplasma gondii* in cats from Colombia, South America and genetic characterization of *T. gondii* isolates. Veterinary Parasitology. 141:42-47.
